# Expression and Regulatory Mechanisms of MicroRNA in Cholesteatoma: A Systematic Review

**DOI:** 10.3390/ijms241512277

**Published:** 2023-07-31

**Authors:** Karolina Dżaman, Katarzyna Czerwaty, Torsten E. Reichert, Mirosław J. Szczepański, Nils Ludwig

**Affiliations:** 1 Department of Otolaryngology, The Medical Centre of Postgraduate Education, 01-813 Warsaw, Poland; otolaryngologia@brodnowski.pl (K.D.); katarzynaczerwaty@gmail.com (K.C.); 2 Department of Oral and Maxillofacial Surgery, University Hospital Regensburg, 93053 Regensburg, Germany; torsten.reichert@ukr.de (T.E.R.); nils.ludwig@ukr.de (N.L.); 3 Department of Biochemistry, Medical University of Warsaw, 02-097 Warsaw, Poland

**Keywords:** cholesteatoma, microRNA, exosomes, small extracellular vesicles

## Abstract

Cholesteatoma is a temporal bone disease characterized by dysfunctions of keratinocytes. MicroRNAs (miRNAs) are evolutionary conserved noncoding RNAs that regulate mRNA expression. They can be packaged into exosomes and transported to target cells that can be used in the future therapy of cholesteatoma. This study aimed to collect knowledge on the role of miRNAs and exosomal miRNAs in cholesteatoma and was conducted according to the PRISMA guidelines for systematic reviews. Four databases were screened: Pubmed/MEDLINE, Web of Science, Scopus, and the Cochrane Library. The last search was run on the 6th of June 2023. We included full-text original studies written in English, which examined miRNAs in cholesteatoma. The risk of bias was assessed using the Office of Health Assessment and Translation (OHAT) Risk of Bias Rating Tool, modified for the needs of this review. We identified 118 records and included 18 articles. Analyses revealed the downregulation of exosomal miR-17 as well as miR-10a-5p, miR-125b, miR-142-5p, miR34a, miR-203a, and miR-152-5p and the overexpression of exosomal miR-106b-5p as well as miR-1297, miR-26a-5p, miR-199a, miR-508-3p, miR-21-3p, miR-584-5p, and miR-16-1-3p in cholesteatoma. The role of differentially expressed miRNAs in cholesteatoma, including cell proliferation, apoptosis, the cell cycle, differentiation, bone resorption, and the remodeling process, was confirmed, making them a potential therapeutic target in this disease.

## 1. Introduction

Cholesteatoma is a well-demarcated non-neoplastic cystic formation composed of stratified keratinizing squamous epithelium, developing in the temporal bone. In cholesteatoma, the hyperproliferation, migration, and altered differentiation of keratinocytes are observed, which are associated with invasive and destructive growth [1].

The incidence rate of cholesteatoma is estimated at 5–9.2 per 100,000 per year [2,3,4,5,6]. The familial occurrence of cholesteatoma is the basis for investigating the genetic background of the disease [7,8], although a family history in this disease is nevertheless quite rare and can only explain a limited number of cases [9]. To date, the higher incidence of cholesteatoma has been associated with various genetic syndromes characterized by craniofacial anomalies, such as Down syndrome [10,11] or Turner syndrome [12,13]. There is a classic clinical classification of cholesteatoma into two phenotypes: congenital and acquired [14]. Middle ear cholesteatomas can be also classified according to the growth pattern into anterior epitympanic, posterior epitympanic, posterior mesotympanic, two routes (both the pars flaccida and the pars tensa are involved), and undetermined [15].

Four theories of the origin of acquired cholesteatoma were presented, including the theories of invagination, immigration, squamous metaplasia, and basal cell hyperplasia [16]. The growth and proliferation of cholesteatoma are closely linked to the upregulation of growth factors, such as epidermal growth factor (EGF) and keratinocyte growth factor (KGF) and its receptors [17,18,19], and cytokines, including IL-1, IL-6, and tumor necrosis factor α (TNF-α) [20]. Cholesteatoma growth can cause both sensorineural and conductive hearing loss, as well as other symptoms, such as dizziness or facial nerve paralysis, and can lead to other complications that include meningitis, mastoiditis, bacterial labyrinthitis, sigmoid sinus thrombophlebitis, or brain abscess.

Currently, the only effective method of treating cholesteatoma is the complete removal and repair of the damaged middle ear structures, and various surgical accesses are used for this purpose [21,22,23]. Unfortunately, recurrence after surgery is still common, and risk factors for recurrence include young age, cholesteatoma localized to the mastoid, stapes, or incus erosion [3]. It was also observed that surgical outcomes are different when comparing rural and urban cohorts [24]. Therefore, there is an urgent need to develop nonsurgical, alternative treatments based on molecular mechanisms. For this reason, it is very important to explore the cellular and molecular mechanisms underlying the pathogenesis of middle ear cholesteatoma.

MicroRNAs (miRNAs) are among small, evolutionary conserved, noncoding RNAs whose function is to regulate the activity of mRNA expression, thus affecting various biological processes. The importance of miRNA-directed gene regulation is becoming increasingly apparent as more miRNAs and their regulatory targets and functions are discovered. A single miRNA can regulate many targets [25]. MiRNAs can be packaged into exosomes and transported to different cells. This process allows for the transfer of genetic information between cells and can have significant functional implications; therefore, they may be used in the future therapy of cholesteatoma.

The role of certain miRNAs in differentiation, development, apoptosis, and oncogenesis in various cancers has been recognized. However, miRNAs have also been shown to play an important role in the development of various otological disorders, such as progressive sensorineural hearing loss, age-related hearing loss, noise-induced hearing loss, cholesteatoma, schwannomas, and inner ear inflammation [26].

In this systematic review, we collect and analyze the results of all studies on the role of miRNAs and exosomal miRNAs in cholesteatoma pathogenesis and their potential usefulness in the nonsurgical treatment of cholesteatoma.

## 2. Materials and Methods

This systematic review was conducted and written in accordance with the updated 2020 Preferred Reporting Items for Systematic Reviews and MetaAnalyses Statement (PRISMA) [27,28].

The PubMed/MEDLINE, Web of Science, Scopus, and Cochrane Library databases were searched systematically. The last search was initiated on the 6th of June 2023 with the language limited to English. No publication time limits were imposed. The detailed search strategy is presented in Table 1. Subsequently, the automatic duplicate identifier function built in EndNote X9 (Clarivate Analytics, London, UK) was used.

Both types of studies, observational and experimental, were included and assessed. Two reviewers (KD and KC) independently screened the studies by title and abstract. Following this selection, full texts were checked. Studies that met the selection criteria were included (Table 2).

**Figure 1:**
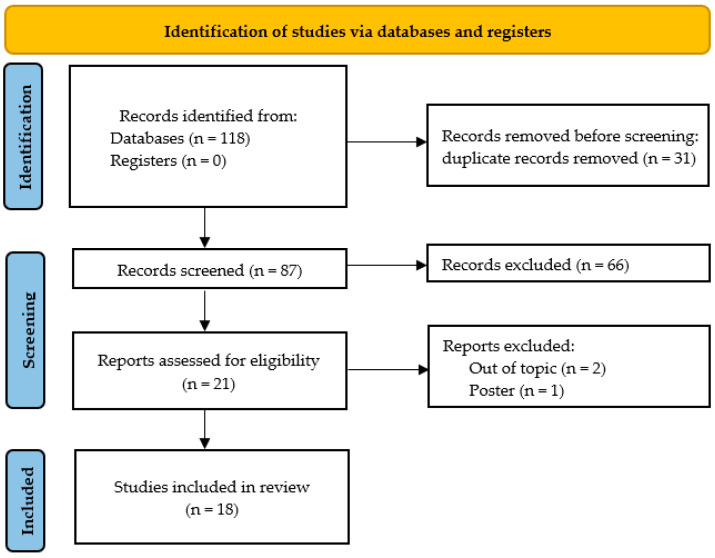
The PRISMA 2020 flow diagram for new systematic reviews, which included searches of databases and registers only.

Only original papers according to the study design were eligible for inclusion. Any apparent discrepancies resulting from the selection process were discussed and resolved by the co-authors (MS and NL or by discussion). Figure 1 provides an overview of the selection process summarized in the PRISMA flowchart.

After assessing the eligibility of all included studies, data for each study were extracted individually by two reviewers. The following results are presented in this review: first author, year of publication, country of origin, the purpose of the study, the miRNAs investigated, and finally the main results on the role of miRNAs in cholesteatoma.

The risk of bias was assessed independently by two authors (KD and KC) using the Office of Health Assessment and Translation (OHAT) Risk of Bias Rating Tool for Human and Animal Studies, modified for the needs of this review [29]. Any disagreement was discussed and resolved by the fourth and fifth co-authors (MS and NL). In general, the studies included were of high to moderate quality. The data are presented in Supplementary Table S2.

## 3. Results and Discussion

### 3.1. Search Results, Study Characteristics, and Study Quality

In total, 118 articles were retrieved through the database search. After applying the search strategy described above, a total of 18 articles were identified that met all inclusion criteria [30,31,32,33,34,35,36,37,38,39,40,41,42,43,44,45,46,47]. A flow diagram of the detailed study selection process is presented in Figure 1.

The articles were analyzed for their basic data and the main results are described in Table S1. The included studies were published between 2009 and 2023, more than half of them in the last five years, reflecting the rapid development in miRNA research in recent years. Almost all of the included studies were conducted in China, except for one study by Friedland et al. [33], which originated in the USA. All included studies used cholesteatoma tissues for analysis. Additionally, the majority of studies included in vitro experiments on cell lines [30,32,34,35,36,37,38,39,42,43,44,46,47]. In the Zheng study [46], a mouse model was also used for in vivo testing.

The quality of the included studies was estimated using the OHAT Risk of Bias Rating Tool for Human and Animal Studies, modified for the needs of this review [29], and it showed that the quality of most studies was intermediate or high. The results are presented in Table S2.

### 3.2. Differentially Expressed MicroRNAs in Acquired Middle Ear Cholesteatoma

In the included studies, miRNA expression profiling analysis was performed between acquired middle ear cholesteatoma and normal skin to identify miRNAs that may be involved in the aetiopathogenesis of middle ear cholesteatoma. Selected miRNAs with significantly increased or decreased expression in cholesteatoma tissue compared to normal skin are shown in Table 3. In Xie’s study [40], the miRNA microarray technology revealed 44 miRNAs with increased expressions (miR-21-3p, miR-584-5p, miR-16-1-3p, etc.) and 175 miRNAs with decreased expressions (miR-10a-5p, miR-152-5p, miR-203b-5p, etc.) in cholesteatoma tissues with a 2-fold change compared to normal skin. Subsequently, qRT-PCR validation showed that miR-21-3p and miR-16-1-3p had significantly higher expressions, while miR-10a-5p showed a markedly reduced expression in middle ear cholesteatoma tissues compared to normal skin. Finally, the Gene Ontology (GO) and Kyoto Encyclopedia Genes and Genomes (KEGG) pathway analyses provided clues that these differentially expressed miRNAs may play an important role in the aetiopathogenesis of middle ear cholesteatoma, including cell proliferation, apoptosis, the cell cycle, differentiation, bone resorption, and the remodeling process.

Differences in miRNA expression may have a potential role in the pathogenesis of cholesteatoma and be a future therapeutic target. The role of some miRNAs has been identified in cholesteatoma and the results of the available studies are presented below. More cholesteatoma research is needed to extend these early discovered relationships.

### 3.3. Role of Exosomal MicroRNAs in Cholesteatoma

#### 3.3.1. MicroRNA-17 Carried by Small Extracellular Vesicles

It was identified that miRNAs may regulate osteoclasts in bone diseases, such as bone loss, metastasis, and osteosarcoma [48]. Furthermore, the role of osteoclasts in bone resorption in cholesteatoma has been confirmed [49,50]. The balance between bone resorption by osteoclasts and bone formation by osteoblasts and the communication between these cells are crucial for bone homeostasis [51,52].

Gong et al. [34] demonstrated that miRNA-17 carried by small extracellular vesicles (sEVs) isolated from keratinocytes of patients with middle ear cholesteatoma can increase receptor activator of nuclear factor-κB ligand (RANKL) expression in fibroblasts and induce osteoclast differentiation. Hereby, RANKL is known to induce osteoclast differentiation [52,53]. Using an in vitro co-culture system consisting of keratinocytes, fibroblasts, and osteoclast precursors, keratinocytes have been shown to stimulate osteoclast differentiation through the induction of RANKL in fibroblasts [52]. Gong et al. [34] observed that fibroblasts treated with keratinocyte-derived sEVs (Ker-Exo) from cholesteatoma patients increased RANKL expression and promoted osteoclast differentiation, and this was dependent on the sEV component miRNA-17, which was downregulated in Ker-Exo in cholesteatoma. These new findings provide a foundation for further research into the effect of miRNAs carried by sEVs on osteoclast differentiation in cholesteatoma, which may be used in the future to treat cholesteatoma and reduce bone destruction in this disease.

#### 3.3.2. MicroRNA-106b-5p Carried by Small Extracellular Vesicles

Proliferating tissue in cholesteatoma requires an increased blood supply; therefore, angiogenesis appears to be a necessary condition for cholesteatoma expansion. The presence and distribution of blood vessels in cholesteatoma were investigated and revealed the presence of increased vascularization in the subepithelial connective tissue of cholesteatoma (perimatrix), which probably plays a role in maintaining continued abnormal growth [54,55,56]. Analysis of the angiogenic growth factors in middle ear cholesteatoma revealed their altered expression compared to middle ear mucosa or auditory meatal skin [55,57,58,59,60,61,62,63]. Therefore, the possibility of using antiangiogenic molecules in the adjuvant treatment of cholesteatoma surgery has been proposed [62].

Moreover, it was confirmed that miRNAs carried by sEVs can participate in stimulating angiogenesis [64,65,66]. Therefore, miRNAs carried by sEVs were one of the factors studied in cholesteatoma angiogenesis. sEVs (size range of 30–150 nm) released into the extracellular space carry cell-specific cargos of proteins, lipids, and nucleic acids (including miRNAs) that can be transferred and participate in intercellular communication [67]. One source of miRNAs in human cholesteatoma is perimatrix fibroblasts (hCPFs), which stimulate microvascular endothelial cells to proliferate and migrate and alter their gene expression patterns by releasing various angiogenic factors [37]. Li et al. [37] were the first to study the function of hCPF-derived sEVs (hCPF-Exo). Their results indicate that hCPF-Exo transports low-expressed miR-106b-5p into endothelial cells and promotes angiogenesis by overexpressing angiopoietin 2.

In conclusion, to develop effective antiangiogenic therapies, it is necessary to understand the mechanisms underlying abnormal angiogenesis.

### 3.4. Other MicroRNAs in Cholesteatoma

#### 3.4.1. MicroRNA-21

The main miRNA investigated in cholesteatoma is miR-21. Its expression is altered in many cancers and other pathologies, including cardiomyopathies [68]. The upregulation of miR-21 in colorectal cancer was shown to be associated with the downregulation of phosphatase tension homolog (PTEN) protein expression, which affects proliferation, antiapoptosis, cell cycle progression, and the invasion of colorectal cancer cells [69]. Furthermore, the tumor suppressor PTEN modulates cell cycle progression and cell survival and its expression was found to be significantly lower in cholesteatoma [70,71], which may account for the impaired inhibition in cholesteatoma. Moreover, programmed cell death 4 (PDCD4) plays a role in the pathogenesis of many cancers, including skin cancer [72,73,74]. However, IL-6 and signal transducers and activators of transcription 3 (STAT3) are upstream activators of miR-21, whose role in cholesteatoma epithelial hyperproliferation has also been confirmed [71,75].

Some investigators [33,76] observed a more than 4-fold higher expression of miR-21 and a reduction in the downstream targets of miR-21, PTEN, and PDCD4 in cholesteatoma compared to normal skin. Chen et al. [31] confirmed these results and additionally reported that miR-21 was particularly elevated in pediatric patients, potentially leading to greater tumor cell proliferation and cholesteatoma invasion, which is reflected in clinical practice, where the disease process is more aggressive and invasive in children than adults [77]. Moreover, PTEN, PDCD4, and high-mobility group AT-hook 2 (HMGA2) protein levels were significantly decreased in pediatric versus adult cholesteatoma patients [31].

Others [30] provided pieces of evidence that miR-21 promotes the proliferation and invasion of cholesteatoma keratinocytes. The number of proliferative cholesteatoma keratinocytes increased after transfection with miR-21 mimics, compared to miRNA controls and miR-21 inhibitors, indicating that miR-21 can promote the growth of cholesteatoma keratinocytes. The inhibitory effect of miR-21 on the apoptosis of cholesteatoma keratinocytes was shown by changing the percentage of apoptotic cells—decreasing it after transfection with miR-21 mimetics and increasing it after transfection with miR-21 inhibitors. The percentage of migrated cholesteatoma keratinocytes transfected with miR-21 mimics was 4.82 times higher when compared to cells transfected with miR-21 inhibitors, indicating that miR-21 promotes the migration and invasion of cholesteatoma keratinocytes.

In a recent study, Chen et al. [32] demonstrated that miR-21 inhibition to some extent slows keratinocyte proliferation and induces the apoptosis of cholesteatoma keratinocytes by inducing cell cycle arrest in the G0/G1 phase through a mechanism associated with the negative regulation of PTEN and PDCD4 expression. Specifically, it was sequentially demonstrated that the proliferation of CK cells (cultured keratinocytes) in the miR-21 inhibition group was significantly lower than in the negative and blind control group, the percentage of CK cells in the G0/G1 phase in the miR-21 inhibition group was significantly higher than in the negative and blind control groups, and protein and mRNA expression levels of PTEN and PDCD4 in CK in the miR-21 group were significantly higher than in the negative and blind control groups.

To sum up, the results of the above studies on cholesteatoma showed a higher level of miR-21 expression, which was associated with the downregulation of two cholesteatoma suppressors, PTEN and PDCD4, which was particularly noticeable for pediatric cholesteatoma. Therefore, the researchers point to the potential use of miR-21 as a therapeutic target in the disease.

#### 3.4.2. MicroRNA-508-3p and Hsa_circ_0000007

Circular RNAs (circRNAs) are covalently closed RNA molecules, which are stable, conserved, and expressed in a tissue-specific manner [78]. CircRNA has been discovered to play a role in the pathogenesis of many cancers [78,79], along with other diseases, such as cardiovascular [80], renal [81], and skin diseases [82], diabetes mellitus [83], and neurological and neuropsychiatric disorders [84,85]. The circRNA expression profile was examined in cholesteatoma and revealed 101 upregulated and 254 downregulated circRNAs in cholesteatoma [86]. CircRNAs can regulate gene expression (transcription and translation) and act as miRNA sponges [79].

It was demonstrated that the increased expression of miR-508-3p in cholesteatoma tissues and cells is inversely correlated with hsa_circ_0000007 expression [38]. Hsa_circ_0000007 expression was significantly lower in cholesteatoma than in normal skin. miR-508-3p appeared to be the targeted miRNA downstream of hsa_circ_0000007; it was upregulated in cholesteatoma, and there was a statistically negative correlation between miR-508-30 and hsa_circ_0000007 in cholesteatoma tissue. The miR-508-3p inhibitor elevated the level of pro-apoptotic factor B-cell lymphoma-2 (Bcl-2), increased PTEN expression, and impeded class I phosphoinositide 3-kinase (PIK3) and protein kinase B (Akt) expressions. Therefore, the miR-508-3p mimic decreased PTEN expression, increased the expression of PIK3 and phosphorylated Akt (p-Akt), and promoted the proliferation of middle ear cholesteatoma cells. The PIK3/Akt signaling pathway plays a key role in cell growth, survival, proliferation, metabolism, and motility in various cancer phenotypes and PIK3/Akt signaling pathway inhibitors are a highly effective treatment strategy [87,88].

In conclusion, the findings point to the targeted regulation of the PTEN/PI3K/Akt signaling pathway by miR-508-3p in the cholesteatoma pathogenesis, while miR-508-3p overexpression in cholesteatoma is probably mediated by the regulation of upstream hsa_circ_0000007.

#### 3.4.3. Let-7a MicroRNA

Let-7a miRNA is categorized as a tumor suppressor and targets oncogenes, including the HMGA2 [89,90]. However, in some rare cases, let-7a miRNA can also act as an oncogene, increasing cancer invasion and chemoresistance [91]. Furthermore, its expression is also altered in cardiovascular diseases and inflammatory processes [92].

The level of let-7a miRNA was significantly elevated in cholesteatoma tissue compared to normal skin, particularly in pediatric patients [31]. HMGA2 protein levels were significantly decreased in pediatric versus adult cholesteatoma patients, which may correspond to an increased apoptosis of keratinocytes and decreased proliferation of cholesteatoma cells [31].

It was observed that let-7a miRNA inhibited the growth of cholesteatoma keratinocytes by reducing keratinocyte proliferation by promoting cell cycle arrest in the G0/G1 phase and inducing cell apoptosis. The authors demonstrated also that let-7a miRNA induces the cell apoptosis, migration, and invasion of cholesteatoma keratinocytes. Finally, let-7a miRNA downregulated miR-21 expression in cholesteatoma keratinocytes, which was also confirmed by the upregulation of miR-21 in the cholesteatoma keratinocytes transfected with the let-7a inhibitor.

To sum up, the results of those studies indicate that let-7a plays a positive role in inhibiting cholesteatoma and can control the proliferation and apoptosis of cholesteatoma keratinocytes through both miR-21 and HMGA2 downregulation.

#### 3.4.4. MicroRNA-125 and Circ_0074491

Another miRNA whose role has been explored in cholesteatoma pathogenesis is miE-125. It has been linked to the regulation of tumorigenesis and tumor development, and its downstream targets include transcription factors such as STAT3, cytokines such as IL-6 and TGFβ, and suppressor protein p53 [93,94]. MiR-125b was found to be significantly downregulated in psoriatic skin, which may contribute to the extensive proliferation and inappropriate differentiation of keratinocytes observed in this disease [95,96]. STAT3 target genes are involved in proliferation, survival, self-renewal, invasion, and angiogenesis [97,98]. The blocking of the JAK2/STAT3 signaling pathway inhibits IL-17-induced vascular endothelial growth factor (VEGF) expression, leading to the inhibition of angiogenesis in inflammatory skin diseases [99]. The expression of STAT3 was found to be significantly higher in the cholesteatoma epithelium than in the normal epithelium of the external auditory canal or retroauricular skin, indicating its potential role in the mechanisms of hyperproliferation and growth in cholesteatoma [100,101].

Zang et al. [44] observed downregulation of miR-125b and upregulation of STAT3, cyclin D1, survivin, and VEGF in cholesteatoma tissues, which is consistent with the results of previous studies [17,57,102,103]. These reports imply that the inhibition of miR-125b expression in cholesteatoma may contribute to high proliferation and low apoptosis through an increased expression of STAT3, which could be used as a potential therapeutic target for intratympanic pharmacotherapy of cholesteatoma [17].

Hu et al. [35] presented the results of a study in which circ_0074491 was defined as a decoy for miR-22-3p and miR-125a-5p in cholesteatoma keratinocytes and was downregulated in cholesteatoma specimens. Furthermore, circ_0074491 knockdown reduced cell cycle arrest and apoptosis; facilitated cell proliferation, migration, colony formation, and the invasion of cholesteatoma keratinocytes; and activated the PIK3/Akt pathway via miR-22-3p and miR-125a-5p in cholesteatoma keratinocytes. Moreover, both miR-22-3p and miR-125a-5p inhibitors reversed the impacts of circ_0074491 silencing on the proliferation, apoptosis, migration, and invasion of cholesteatoma keratinocytes. In conclusion, circ_0074491 was shown to regulate cholesteatoma progression through the PIK3/Akt pathway and binding to miR-22-3p and miR-125a-5p.

Finally, most researchers confirmed the protective role of miR-125 in cholesteatoma, especially through the impact on the apoptosis pathway, and found it to be significantly downregulated in patients with this disease.

#### 3.4.5. MicroRNA-10a-5p

MiR-10a-5p is another miRNA that plays a role in cancer and was studied in cholesteatoma. A lower expression of miR-10a-5p has been linked to the progression of hepatocellular carcinoma [104], melanoma [105], and ovarian cancer [106]. However, miR-10a-5p was also found to be upregulated in the affected skin of atopic dermatitis patients and in proliferating keratinocytes [107]. Xie et al. [40] identified the phosphatidylinositol-4,5-bisphosphonate 3-kinase catalytic subunit α (PIK3CA) as an important miR-10a-5p target gene. PIK3CA is a catalytic subunit of PIK3 that plays a role in the EGFR/PIK3/Akt/cyclinD1 signaling pathway, which is active in cholesteatoma and may have a key function in epithelial hyperproliferation [17].

In another study [41], middle ear cholesteatoma tissues showed significantly decreased miR-10a-5p expression and increased PIK3CA expression compared to normal posterior ear skin tissues (*p* < 0.05). The results of target gene prediction revealed that PIK3CA may be an miR-10a-5p target gene. Additionally, miR-10a-5p and PIK3CA expression levels were significantly negatively correlated in middle ear cholesteatoma tissues (r = −0.926, *p* < 0.001). These results indicate that miR-10a-5p can inhibit cholesteatoma proliferation and differentiation through the negative regulation of its target gene, PIK3CA.

#### 3.4.6. MicroRNA-802

MiR-802 is another miRNA that has been studied in cholesteatoma. Previously, its role was confirmed in mediating the pathogenesis of a variety of tumors [108], obesity [109,110], osteoporosis [111], and inflammatory bowel disease [112].

Li et al. [36], for the first time, demonstrated the role of the NF-κB/miR-802/PTEN signaling pathway in cholesteatoma. In this study, cholesteatoma tissues showed a high activation of NF-κB and upregulation of proinflammatory cytokines such as TNFα, IL-1b, and IL-6. The researchers observed the upregulation of miR-802 in keratinocytes treated with TNFα, IL-1b, or IL-6 and revealed that the miR-802 promoter contained a functional NF-κB/P65 binding site. The authors demonstrated that miR-802 can promote keratinocyte proliferation and cell cycle progression. They also noticed that keratinocytes transfected with an has-miR-802 mimetic showed a significantly increased proliferation rate, a significantly reduced percentage of cells in the G1/G0 phase, and an increased percentage of cells in the S phase. Additionally, the inhibition of miR-802 reduced the above effects. Finally, the authors performed a computational analysis using miRWalk software and found that the gene encoding PTEN harbored an miR-802 binding site and suggested that miR-802 could directly inhibit PTEN expression by targeting its 30-untranslated region (UTR).

#### 3.4.7. MicroRNA-1297, MicroRNA-26a-5p, and MicroRNA-203a

MiR-1297 and miR-26a-5p are other miRNAs that have been studied in cholesteatoma and are widely investigated in oncology. The inhibitory effect of miR-1297 on cancer progression has been observed in various malignant entities [113,114,115,116,117,118,119,120,121,122,123,124]. In addition to its role in tumorigenesis, miR-26a-5p similarly plays an important role in pulmonary fibrosis [125] and Alzheimer’s disease [126]. However, the included studies provided new information on the role of miR-1297 and miR-26a-5p concerning the pathogenesis of cholesteatoma. miR-1297 and miR-26a-5p were shown to inhibit the progression of cholesteatoma keratinocytes by targeting the B-cell-specific Moloney murine leukemia virus insertion site 1 (BMI1) [47]. BMI1 is a component of the polycomb 70 repressive complex 1, which is a transcriptional repressor that increases cell survival [127]. The role of BMI1 has been identified in various cancers [128,129,130,131,132,133] and was shown to be linked to poor prognosis. Additionally, BMI1 levels were found to be elevated in transformed keratinocytes, skin tumors, and psoriasis and were shown to play a role in keratinocyte survival [127,134]. It was noted that BMI1 is expressed mainly in the basal and suprabasal layers of the epithelium in normal skin [43,134,135], but in cholesteatoma it was found in almost all layers of the epithelium, and the intensity of staining was comparably stronger [43].

A significantly elevated mRNA expression of BMI1 and protein level of BMI1 were demonstrated in cholesteatoma tumor tissues relative to normal retroauricular skin specimens [43,47]. The downregulation of BMI1 was shown to significantly inhibit the proliferation of human immortalized keratinocytes, indicating a pro-oncogenic role for BMI1 in cholesteatomas, which is consistent with observations in other malignant entities [47]. The prediction result obtained with DianaTools software showed that BMI1 directly interacts with miR-1297 and miR-26a-5p in human immortalized keratinocytes.

Another miRNA targeting BMI1 is miR-203a. The role of miR-203 as a tumor suppressor has been demonstrated in a number of cancers [136,137,138]. It is known that miR-203 promotes epidermal differentiation by limiting the proliferative potential [139] and is upregulated in psoriatic lesions [95], while the upregulation of miR-203 expression by oleic acid treatment accelerates human keratinocyte differentiation [140]. Therefore, its role in the growth and proliferation of cholesteatoma was investigated [43], leading to the observation that miR-203a is downregulated and BMI1 upregulated in cholesteatoma. Subsequently, the authors demonstrated in the dual-luciferase reporter assay (DLRA) that BMI1 is a direct target of miR-203a. Furthermore, reduced miR-203a expression increased BMI1 expression; promoted the proliferation, colony formation, and migration of HaCaT cells (a spontaneously transformed aneuploid immortal keratinocyte cell line from adult human skin); and inhibited apoptosis. Moreover, p-Akt was significantly increased in cholesteatoma tissues and was positively correlated with BMI1, which is consistent with other studies [141,142]. Finally, BMI1 suppression decreased p-Akt expression in HaCaT cells and subsequent miR-203a inhibition reversed this effect [43].

Taken together, all the studies confirmed that all three miRs, miR-1297, miR-26a-5p, and miR-203a, inhibit the progression of cholesteatoma keratinocytes by targeting BMI1. The new reports reveal the need for further research to explore specific therapeutic methods for cholesteatoma patients through a combination of nanotechnology and molecular drugs targeting those miRNAs or BMI1.

#### 3.4.8. MicroRNA-199a

MiR-199a was shown to be involved in the proliferation, migration, invasion, and apoptosis of cancer cells from different primary tumors [143]. Its role has also been confirmed in other noncancerous diseases, such as generalized epilepsy [144] or osteoporosis [145].

Yao et al. [42] reported that miR-199a was significantly upregulated in cholesteatoma tissues compared to the normal retroauricular skin tissues, which facilitated the proliferation, migration, and invasion of cholesteatoma keratinocytes in vitro, while miR-199a downregulation caused the opposite effects. The authors demonstrated a negative regulatory relationship between miR-199a and PNRC1 and found miR-199a binding to the 3′-UTR of PNRC1.

To sum up, the upregulation of miR-199a in cholesteatoma potentially plays a role in the development of cholesteatoma and could be used as a biomarker or therapeutic target.

#### 3.4.9. MicroRNA-142-5p

Another study focused on the role of miR-142 in cholesteatoma pathogenesis. miR-142 is expressed in many tissues and was found to play an important role in inflammatory and immune responses [146,147,148,149]. The differential expression patterns of miR-142-5p were observed in cancer tissues [150,151,152,153,154]. A downstream target gene of miR-142-5p is cyclin-dependent kinase 5 (CDK5) [39], whose role has been found in the development and progression of many cancers [155,156,157] and neurodegenerative [158,159] and inflammatory disorders [160,161,162]. Sui et al. [39] demonstrated that miR-142-5p directly inhibits the CDK5-mediated upregulation of inflammatory cytokines in acquired middle ear cholesteatoma, making it a potential therapeutic target in this disease.

#### 3.4.10. Micro-RNA-34

MiR-34 is characterized by antitumor properties, is induced by the tumor suppressor p53, and can be used to control tumor progression [163,164]. Thus, one of the included studies revealed the function of miR-34 in cholesteatoma [42]. The researchers conducted an experimental study using nanoparticles in which therapeutic targets were achieved by regulating miR-34a expression. They showed that free rubone and rubon-containing drug nanoparticles (RC NPs) can significantly inhibit the proliferation and migration ability of cholesteatoma cells in children’s middle ear cholesteatoma. The authors demonstrated that drug-loaded nanoparticles could deliver rubone into cells and upregulate miR-34a levels and downregulate mRNA expression levels of Bcl-2, CDK6, and cyclin D1 in cells. The expression level of miR-34a in the free rubone group was slightly higher than that in the RC NPs group, which was due to the slow and controlled release of nanoparticles. The study indicated the potential of these novel forms of treatment in both cholesteatoma and also other oncologic diseases.

### 3.5. Strengths and Limitations of the Included Studies

Research on the association between cholesteatoma and microRNAs is still in the exploratory phase. The main advantage of the included studies is that they provide discoveries in the field of understanding the etiopathogenesis of cholesteatoma, which may open up the possibility of developing new microparticle-based treatment strategies for this disease. It should be noted that some of the studies included cholesteatoma samples collected in the pediatric population, which also provide better insight into the pathogenesis of this more aggressive form of the disease [31,32,46]. Moreover, new studies involving miRNAs carried by sEVs were also among the included studies, which provided new insight into the role of these nanoparticles in cholesteatoma [34,37].

However, many of the included studies analyzed a small number of clinical samples (most studies include cholesteatoma tissues from less than 30 patients), and the population from which tissues were collected was not homogeneous in terms of age or other parameters. In some studies, it was not specified precisely whether the samples were only from acquired cholesteatoma patients. Another limitation is the lack of information on the number of people from whom the tissues analyzed in some studies were collected [32,33,34,37]. Future studies should use a larger number of samples and ensure the homogeneity of the groups to avoid factors that could affect the results of the study. The pathogenesis of acquired and congenital cholesteatoma can differ, so it is important to emphasize which form of this disease the study is concerned with. Larger, more homogenized cohorts would provide more robust and generalizable data on differentially expressed miRNAs. Another problem is the limited validation in the included studies. Many findings are based on initial microarray screens that have not been extensively replicated in independent datasets. Further validation is needed to confirm key microRNAs and their roles. Unfortunately, microRNA mechanisms of action in promoting or inhibiting cholesteatoma progression are still unclear, and further work is needed to clarify how they affect keratinocyte behaviors.

Retroauricular skin has been used as a control material in many of the included studies [31,33,35,36,37,38,40,41,42,43,44,45,47]. There are reports that the selection of control group material in cholesteatoma studies can be important for the outcome of the studies, with deep meatal skin specimens being more similar in protein profile to cholesteatoma than retroauricular skin [165,166]. Moreover, canal skin is exposed to infection, which can stimulate cytokine expression and changes in protein expression, which can also affect research results [33].

Most studies show only correlational analyses established between miRNA levels and target mRNAs/proteins. Experimental manipulations are often lacking to demonstrate causality.

The study by Zhu et al. [47] provides new insights into the involvement of miR-1297/BMI1 and miR-26a-5p/BMI1 regulatory mechanisms in the pathogenesis of cholesteatoma, but only in vitro experiments were performed, which have some limitations. Animal studies are needed to confirm the relationships discovered.

The study by Li et al. [37] has its limitations because it is an in vitro study in which miR-106b-5p carried by sEVs derived from cholesteatoma hCPFs is just one of the sEV miRNA cargo components that promote angiogenesis in middle ear cholesteatoma, and the possible influence of other pro-angiogenic factors of the sEV cargo cannot be ignored. Therefore, further studies should be performed in an animal model of middle ear cholesteatoma to investigate the suppressive effect of sEV-associated miR-106b-5p on cholesteatoma growth in vivo. Animal models could help determine the true significance of microRNAs in cholesteatoma pathogenesis.

Recent studies suggest that abnormal miRNA expression may contribute to cholesteatoma pathogenesis, though causal links remain unclear based on preliminary evidence. Targeting relevant microRNAs could potentially offer adjunctive treatments to supplement current surgical treatments like type 1 tympanoplasty [167,168]. However, microRNA-based therapies for cholesteatoma remain in the early stages of research, with limited validation in large cohorts and animal models. Further rigorous investigation is needed before these approaches could mature sufficiently to realistically improve outcomes of procedures like type 1 tympanoplasty in clinical practice.

## 4. Conclusions

Cholesteatoma is a disease that significantly impairs function, and its complications can be life-threatening. The pathogenesis of this disease is still under investigation, and knowledge in this field has recently been supplemented by studying the role of certain miRNAs and circRNAs. These are very important discoveries as they might contribute to the identification of pharmacological targets for the treatment of cholesteatoma, but there is a need for further validation and causative evidence. The presence of miRNAs in exosomes suggests a mechanism by which cells can communicate and modulate gene expression in a paracrine or endocrine manner. However, further large-scale validation studies, the experimental manipulation of microRNA levels, the elucidation of mechanisms, and in vivo testing are needed to substantiate these initial correlations and guide potential therapeutic applications. Comprehensive microRNA profiling in larger cohorts to identify robust disease-associated miRNAs are needed. Furthermore, future research should include integrated analyses of miRNAs, target mRNAs, and proteins to map regulatory networks. Moreover, the specific mechanisms by which miRNAs regulate cholesteatoma development remain largely unknown. Future work should contain functional studies manipulating microRNA levels and assessing impacts on keratinocyte behaviors and clarify how miRNAs influence keratinocyte proliferation, apoptosis, differentiation, etc. Additionally, there is limited functional testing in vitro and a lack of in vivo animal models to determine the true significance of miRNAs in disease pathogenesis. Investigations of miRNA roles in growth factor and cytokine signaling dysregulation are required. Finally, future work should also consider the development and testing of miRNA-based therapies in animal models. Hopefully, in the future, RNA- and protein-based therapies using nanoparticles will be possible for the nonsurgical or adjunctive treatment of cholesteatoma.

## Figures and Tables

**Table 1:** Databases used and corresponding search lines.

Databases	Number of Hits	Search Lines
*Scopus*	83	TITLE-ABS-KEY ((microRNA) OR (miRNA)) AND (cholesteatoma) Limited to 1. Document type: Article 2. Language: English
*Web of Science*	18	(AB = ((microRNA) OR (miRNA))) AND (AB = (cholesteatoma))
*Pubmed/Medline*	17	(“Cholesteatoma” [Mesh]) AND “MicroRNAs” [Mesh]
*Cochrane*	0	#1 “cholesteatoma” [Mesh] #2 “microRNAs” [Mesh] #1 AND #2

**Table 2:** Inclusion and exclusion criteria for the studies included in the review.

	**Inclusion Criteria**	**Exclusion Criteria**
*Topic*	Studies concerning miRNA/cholesteatoma	Studies not related to miRNA/cholesteatoma
*Study type*	Original articles	Reviews, case reports, book chapters, expert opinions, letters to the editor, conference reports, posters
*Study status*	Completed, published	Unfinished, unpublished
*Language*	Full text available in English	Language other than English or only abstract available in English
*Quality*	Good-quality research studies	Poor-quality research studies

**Table 3:** Differentially expressed miRNAs in cholesteatoma tissue.

Study	miRNA	Expression in Cholesteatoma Tissue
Yang, J. [41]	miR-10a-5p	downregulated
Zhu, X. [47]	miR-1297, miR-26a-5p	downregulated
Zang, J. [44]	miR-125b	downregulated
Sui, R. [39]	miR-142-5p	downregulated
Yao, L. [42]	miR-199a	upregulated
Liu, D. [38]	miR-508-3p	upregulated
Hu, Y. [35]	miR-22-3p, miR-125a-5p	upregulated
Gong, N. [34]	exosomal miR-17	downregulated
Zang, J. [43]	miR-203a	downregulated
Xie, S. [40]	miR-21-3p, miR-584-5p, miR-16-1-3p, miR-338-5p, miR-320b, miR-181a-3p, miR-181a-5p, miR-181b-5p, miR-335-3p, miR-155-5p, miR-224-3p, etc.	upregulated
	miR-10a-5p, miR-152-5p, miR-203b-5p, miR-30a-5p, miR-1297, miR-539-3p, miR-9-3p, miR-769-3p, etc.	downregulated
Li, Y. [37]	exosomal miR-106b-5p	downregulated
Chen, X. [31]	miR-21, let-7a miRNA	upregulated
Friedland, D.R. [33]	miR-21	upregulated

## Data Availability

Not applicable.

## Supplementary Materials

The following supporting information can be downloaded at: https://www.mdpi.com/article/10.3390/ijms241512277/s1.
