# Supplementary material for: Expression and Regulatory Mechanisms of MicroRNA in Cholesteatoma: A Systematic Review
Source: Int J Mol Sci. 2023 Jul 31;24(15):12277. doi: 10.3390/ijms241512277 (PMC10418341; doi:10.3390/ijms241512277)
Supplement: Supplementary file 1 [file ijms-24-12277-s001.zip › ijms-2522848-supplementary.pdf]

Table S1. Characteristics of included studies covering the main findings concerning the role of miRNAs in cholesteatoma.

Study	Year	MiRNA	Main aim	Relevant Results
Yang, J. [38]	2023	miR-10a-5p	to explore the role of miR-10a-5p and the PIK3CA in the pathogenesis of middle ear cholesteatoma	• middle ear cholesteatoma tissues showed significantly decreased miR-10a-5p expression and increased PIK3CA expression than normal posterior ear skin tissues ($p < 0.05$)<br>• miR-10a-5p and PIK3CA expression levels were significantly negatively correlated in middle ear cholesteatoma tissues ($r = -0.926$, $p < 0.001$)
Zhu, X. [44]	2022	miR-1297, miR-261-5p	to explore the biological role and underlying mechanisms of BMI1 in the progression of cholesteatoma	• BMI1 showed high expression in cholesteatoma tumor tissues<br>• BMI1 knockout could inhibit proliferation, colony formation, migration, invasion, and cell cycle progression and promote keratinocyte apoptosis<br>• BMI1 was a target of miR-1297 and miR-26a-5p, and the addition of BMI1 could abolish the suppressive effect of miR-1297 or miR-26a-5p overexpression on the malignant behavior of keratinocyte cells.
Zang, J. [41]	2022	miR-125b	to study the expression and regulatory mechanisms of miR-125b and its downstream genes in cholesteatoma	• downregulation of miR-125b and upregulation of STAT3, cyclin D1, survivin, and VEGF in cholesteatoma tissues
Sui, R. [36]	2022	miR-142-5p	to investigate the roles of miR-142-5p and CDK5 in inflammatory responses in acquired middle ear cholesteatoma	• the expression of miR-142-5p was significantly reduced in acquired middle ear cholesteatoma and was negatively correlated with the expression of CDK5<br>• miR-142-5p can inhibit CDK5 expression by directly targeting 3' UTR of CDK5
Zheng, H. [43]	2021	miR-34a	to investigate the effect of nanodelivery of the miR34a regulator, rubine, on proliferation, apoptosis, and migration ability in children with middle ear cholesteatoma	• RC NPs can significantly inhibit the proliferation and migration ability of children's middle ear cholesteatoma cells by increasing miR-34a levels and downregulating mRNA expression of Bcl-2, CDK6, and cyclin D1 in the cells
Yao, L. [39]	2021	miR-199a	to investigate the expression and role of miR-199a in cholesteatoma	• upregulation of miR-199a was found in cholesteatoma tissues, which facilitated the proliferation, migration, and invasion of HaCaT cells<br>• miR-199a downregulation caused the opposite effects.

Liu, D. [35]	2021	miR-508-3p	to investigate the regulatory effect of miR-508-3p on the proliferation and apoptosis of middle ear cholesteatoma cells and to explore the underlying regulatory mechanism	• miR-508-3p expression was upregulated in cholesteatoma tissues and cells and inversely correlated with hsa_circ_0000007 expression<br>• overexpression of miR-508-3p promoted the proliferation of cholesteatoma cell<br>• in the DLRA, PTEN was a direct target of miR-508-3p<br>• rescue assays confirmed that PTEN could reverse the effect of miR-508-3p overexpression on cell proliferation
Hu, Y. [32]	2021	miR_22-3p, miR-125a-5p	to explore the role of circ_0074491, miR-22-3p, and miR-25a-5p in cholesteatoma	• circ_0074491 was downregulated in cholesteatoma tissues<br>• circ_0074491 knockdown facilitated cell proliferation, migration, invasion, and inhibited cell apoptosis, and activated the PIK3/Akt pathway in cholesteatoma keratinocytes via miR-22-3p and miR-125a-5p<br>• identification of circ_0074491 as a decoy for miR-22-3p and miR-125a-5p in cholesteatoma keratinocytes<br>• both miR-22-3p and miR-125a-5p silencing reversed the impacts of circ_0074491 silencing on proliferation, apoptosis, migration, and invasion of cholesteatoma keratinocytes
Chen, X. [29]	2021	miR-21	to investigate the effects of miR-21 on glial cell proliferation in children with cholesteatoma, as well as related mechanisms	• CK cell proliferation in the miR-21 inhibition group was significantly lower than in the negative and blind control groups ($p < 0.05$)<br>• the percentage of CK cells in the G0/G1 phase in the miR-21 inhibition group was significantly higher than in the negative and blind control groups ($p < 0.05$)<br>• protein and mRNA expression levels of PTEN and PDCD4 in CK in the miR-21 group were significantly higher than in the negative and blind control groups ($p < 0.05$)
Gong, N. [31]	2020	miR-17	to investigate the effects of Ker-Exo on osteoclast differentiation by co-culturing Ker-Exo with fibroblasts and osteoclast precursor cells	• Ker-Exo primed fibroblasts upregulated RANKL expression and promoted osteoclast differentiation<br>• the effect of Ker-Exo depended on its miRNA-17 component<br>• miRNA-17 was downregulated in Ker-Exo and may increase RANKL level in fibroblasts, thereby promoting osteoclast differentiation
Zang, J. [40]	2018	miR-203a	to explore the regulatory mechanisms of miR-203a and BMI1 in cholesteatoma	• downregulation of miR-203a and upregulation of BMI1 in cholesteatoma<br>• in the DLRA, BMI1 was a direct target of miR-203a<br>• silencing of miR-203a increased BMI1 expression, promoted proliferation, colony formation, and migration of HaCaT cells, and inhibited apoptosis

				• p-Akt was significantly increased in cholesteatoma tissues and was positively correlated with BMI1<br>• suppression of BMI1 decreased p-Akt expression in HaCaT cells and subsequent miR-203a inhibition reversed this phenomenon
Xie, S. [37]	2018	miR-21-3p, miR-584-5p, miR-16-1-3p, miR-10a05p, miR-152-5p, miR-203b-5p, etc.	to analyze the miRNA expression profiling between acquired middle ear cholesteatoma and normal skin	• the miRNA microarray technology revealed 44 miRNAs with increased expression (miRNA-21-3p, miRNA-584-5p, miRNA-16-1-3p, etc.) and 175 miRNAs with decreased expression (miRNA-10a-5p, miRNA-152-5p, miRNA-203b-5p, etc.) in cholesteatoma tissues with a 2-fold change compared to normal skin<br>• RT-PCR validation showed that miRNA-21-3p and miRNA-16-1-3p had significantly higher expressions, while miRNA-10a-5p showed markedly reduced expression in middle ear cholesteatoma tissues compared to normal skin<br>• the GO and KEGG pathway analyses provided clues that these differentially expressed miRNAs may play an important role in the aetiopathogenesis of middle ear cholesteatoma, including cell proliferation, apoptosis, cell cycle, differentiation, bone resorption, and remodeling process
Li, Y. [34]	2018	miR-106b-5p	to explore whether exosomes derived from hCPFs-Exo can promote angiogenesis	• hCPFs-Exo were able to promote migration and tube formation in HUVECs<br>• hCPFs-Exo with low miR-106b-5p expression were transferred into HUVECs, and reduced miR-106b-5p expression could promote angiogenesis by targeting Angiopoietin 2 through binding to its 30-UTR<br>• low levels of miR-106b-5p triggered Angiopoietin 2 overexpression and significantly increased HUVECs migration and tube formation.
Chen, X. [27]	2016	miR-21	to investigate the role of miR-21 in the control of proliferation, apoptosis, and invasion in cholesteatoma keratinocytes	• the number of proliferative EdU-positive cholesteatoma keratinocytes and TUNEL-positive cells increased in cholesteatoma keratinocytes transfected with miR-21 mimetics<br>• the number of migrating cells transfected with miR-21 mimetics was higher compared to migrating cells transfected with miR-21 inhibitors or control miRNAs
Zhang, W. [42]	2015	miRNA let-7a	to investigate the functions of let 7a miRNA in cholesteatoma keratinocytes using let 7a	• let-7a miRNA inhibited the growth of cholesteatoma keratinocytes by reducing keratinocyte proliferation by promoting cell cycle arrest in the G0/G1 phase and inducing cell apoptosis; Let-7a miRNA inhibited migration and invasion of cholesteatoma keratinocytes<br>• let-7a miRNA downregulated miR-21 expression

			miRNA mimics and a let 7a inhibitor	
Li, N. [33]	2014	miR-802	to investigate the role of NF-κB/miR-802/PTEN signaling pathway in cholesteatoma	• NF-kB inflammatory signaling pathway was strongly activated in cholesteatoma<br>• NF-kB activation increased miR-802 expression<br>• chromatin immunoprecipitation assays showed that P65 could uniquely bind to miR-802 promoter<br>• miR-802 overexpression promoted keratinocyte cell proliferation and cell cycle progression, while miR-802 inhibition reduced these effects<br>• miR-802 directly inhibited PTEN expression by targeting its 30-UTR
Chen, X. [28]	2011	miR-21, miRNA let-7a	investigating the role of miR-21 and let-7a miRa in paediatric and adult cholesteatoma	• miR-21 and let-7a levels were significantly elevated in cholesteatoma tissue compared to normal skin, particularly in pediatric patients<br>• PTEN, PDCD4, and HMGA2 protein levels were significantly decreased in pediatric versus adult cholesteatoma patients
Friedland , D. [30]	2009	miR-21	to characterize and compare microRNA and protein expression in cholesteatoma relative to normal skin	• more than a 4-fold higher expression of miR-21 in cholesteatoma<br>• the downstream targets of miR-21, PTEN, and PDCD4, were found to be reduced in cholesteatoma compared to normal skin

Akt - protein kinase B; Bcl-2 - B-cell lymphoma-2; BMI1 - B-cell-specific Moloney murine leukemia virus insertion site 1; CDK5 - cyclin-dependent kinase 5; CK - cultured keratinocytes; DLRA - dual-luciferase reporter assay; EdU - 5-ethynyl-2’-deoxyuridine incorporation assay; GO - Gene Ontology; HaCaT cells - a spontaneously transformed aneuploid immortal keratinocyte cell *line* from adult human skin; hCPFs-Exo - exosomes derived from human cholesteatoma perimatrix fibroblasts; HMGA2 - high mobility group AT-hook 2 protein; HUVECs - human umbilical vein endothelial cells; KEGG - Kyoto Encyclopedia Genes and Genomes; Ker-Exo - keratinocyte-derived exosomes; NF-kB - nuclear factor kappa-light-chain-enhancer of activated B cells; p-Akt - phosphorylated protein kinase B; PDCD4 - programmed cell death factor-4; PIK3CA - phosphatidylinositol-4,5-bisphosphonate 3-kinase catalytic subunit α; PTEN - phosphatase tension homologue; RANKL - receptor activator of nuclear factor-κB ligand; RC NPs - rubon-containing drug nanoparticles; RT PCR - real-time polymerase chain reaction; STAT3 - signal transducers and activators of transcription 3; TUNEL - terminal deoxynucleotidyl transferase-mediated dUTP-digoxigenin nick-end-labeling; UTR - untranslated region; VEGF - vascular endothelial growth factor

Table S2. The risk of bias studies included in a systematic review assessed using the OHAT risk of bias.

**Study**	Was administered dose or exposure level adequately randomized?	Was allocation to study groups adequately concealed?	Did selection of study participants result in appropriate comparison groups?	Were experimental conditions identical across study groups?	Were the research personnel and human subjects blinded to the study group during the study?	Were outcome data complete without attrition or exclusion from analysis?	Can we be confident in the exposure characterization?	Can we be confident in the outcome assessment?	Were all measured outcomes reported?	Were there no other potential threats to internal validity (e.g., statistical methods were appropriate and researchers adhered to the study protocol)?
Yang, J. [38]	+	+	+	++	−	++	+	++	++	+
Zhu, X. [44]	+	++	+	++	−	++	++	++	++	++
Zang, J. [41]	+	+	+	++	−	++	++	++	++	+
Sui, R. [36]	+	+	+	++	−	++	++	++	++	+
Zheng, H. [43]	+	+	+	++	−	+	++	++	+	++
Yao, L. [39]	+	+	+	++	−	++	+	++	++	+
Liu, D. [35]	+	+	++	++	−	++	+	+	+	+
Hu, Y.	+	+	+	++	−	+	++	++	++	++

[32]										
Chen, X. [29]	+	+	++	+	−	++	+	++	++	++
Gong, N. [31]	+	+	+	++	−	+	++	++	++	+
Zang, J. [40]	+	+	+	++	−	++	+	++	++	+
Xie, S. [37]	+	+	+	+	−	+	+	++	+	+
Li, Y. [34]	+	+	+	++	−	+	++	++	++	++
Chen, X. [27]	+	+	++	+	−	++	+	+	++	+
Zhang, W. [42]	+	+	++	+	−	++	+	++	++	+
Li, N. [33]	+	++	+	++	−	+	++	++	++	++
Chen, X. [28]	+	+	+	+	−	++	+	++	++	+
Friedland, D. [30]	+	++	+	+	−	+	+	++	++	+

++ sign indicates low, + sign indicates probably low, − sign indicates probably high, −− sign indicates high risk of bias
